# Editorial: *Fusarium* pathogenesis: Infection mechanisms and disease progression in host plants

**DOI:** 10.3389/fpls.2022.1020404

**Published:** 2022-10-12

**Authors:** Giovanni Beccari, Guixia Hao, Huiquan Liu

**Affiliations:** ^1^ Department of Agriculture, Food and Environmental Sciences, University of Perugia, Perugia, Italy; ^2^ Mycotoxin Prevention and Applied Microbiology Research Unit, National Center for Agricultural Utilization, United States Department of Agriculture - Agricultural Research Service (USDA-ARS), Peoria, IL, United States; ^3^ State Key Laboratory of Crop Stress Biology for Arid Areas and College of Plant Protection, Northwest A&F University, Yangling, China

**Keywords:** *Fusarium*, virulence, effectors, host defense, host-pathogen interaction

*Fusarium* is one of the most important fungal plant pathogens, which cause severe diseases on numerous crops (Leslie and Summerell, 2013). The diseases reduce crop yield, thereby resulting in economic losses (Nganje et al., 2004; Viljoen et al., 2020). In addition, some *Fusarium* spp. can produce mycotoxins that contaminate, in particular, infected grains and pose a threat to human and animal health (Escrivá et al., 2015). *Fusarium* spp. adopt intricate pathways to suppress plant defenses. The pathogens invade the host and colonize it utilizing various infection strategies. In the *Fusarium* genome, in addition to a region responsible for primary metabolism (core genome), there are regions responsible for pathogen virulence (adaptive genome) (Ma et al., 2013). The understanding of the mechanisms that *Fusarium* uses to overcome host defenses will provide novel targets to control diseases. Therefore, this Research Topic aimed to highlight the recent works on key species of *Fusarium* and their interactions with hosts. This Research Topic attracted 16 manuscripts, of which 8 were accepted and published. The articles cover important outcomes of *Fusarium* pathogenesis, and some key aspects are summarized below.

Genetic exchange in *Fusarium oxysporum* (Fo) can occur *via* horizontal chromosome transfer (HCT), a process involving the transfer of “accessory” chromosomes (Ma et al., 2010). In plant pathogenic strains of Fo, accessory chromosomes that are acquired *via* HCT may have the genes required for host-specific pathogenicity. The review paper of Epstein et al. highlights aspects of the biology of *F. oxysporum* f. sp. *apii* (Foa) race 4 and its interactions with the hosts that could potentially apply to other *Fusarium* pathosystems. In detail, if Foa race 4 and another *forma specialis*, *F. oxysporum* f. sp. *coriandri* (Foci), co-occur in the same host (i.e., coriander) under conditions conducive for either somatic compatibility or conidial anastomosis tube formation, Foa and Foci could perform nuclear transfer and subsequently HCT. This could lead to the development of more virulent genotypes that could elude the defenses of additional plant species and thereby cause more severe economic loss under projected climatic conditions.

Effectors play a key role during fungus-plant interactions. Numerous effectors have been identified in fungal plant pathogens and some of them have been characterized for their virulence functions (Tariqjaveed et al., 2021). Although endophytic and beneficial interactions of Fo with plants are common, there are relatively few investigations of such interactions at the molecular level (Pereira et al., 2019). The molecular basis that determines endophytic or pathogenic lifestyle is still not well understood. In this context, the paper of Constantin et al. proposes that pathogenicity is an exception and is triggered by a specific set of effector genes that expressed when interacted with a susceptible host. Conversely, an endophytic behavior is common for Fo, which may be determined by core effectors.

Interestingly, the paper of Wang et al. describes that the small, secreted protein FoSsp1 stimulates host defenses and negatively controls virulence in *F. oxysporum* f. sp. *cubense* race 4 (Foc4). In general, during the colonization process, vascular wilt fungi, secrete small cysteine-rich proteins to suppress plant immunity (Niu et al., 2021). FoSsp1 could function as a putative elicitor that induces high expression of host defense-associated genes and, therefore, negatively regulates conidiation and virulence of pathogens.

In a similar context, the contribution of Yue et al. describes that transgenic tomato overexpressing a lectin receptor-like kinase, SlLecRK1, provides resistance to *F. oxysporum* f. sp. *radicis-lycopersici* (Forl). The roles of lectin receptor-like kinases (LecRKs) in defensive responses and immunity in many hosts are critical. The authors suggested that LecRKs enahnce the resistance of tomato plants to Forl principally by activating ethylene-responsive transcription factor genes.

Host responses to *Fusarium* infection could also be affected by input made by farmers during cultivation. For example, *Fusarium* wilt of banana (FWB) may be exacerbated by nitrogen (N) fertilizers that are routinely applied during banana cultivation (Segura-Mena et al., 2021). To better understand this effect of N, the contribution of Orr et al. indicates that the host defenses to FWB were influenced by the application of N, particularly in the form of ammonium. The authors revealed that levels of N applied changed the expression of host metabolic pathways that are related to stress response signaling. For instance, a negative correlation between pathogenesis-related protein 1, a well-known marker for biotic stress response, and the rate of ammonium fertilizer was reported by the authors.

Reactive oxygen species (ROS) production is an initial defense reaction during plant-pathogen interactions. Nevertheless, the roles of ROS during the progress of *Fusarium* Head Blight (FHB) of cereals remain unclear. For this reason, the paper of Hao et al. investigated immune responses in wheat triggered by chitin, a major component of fungal cell walls. In wheat rachises and rachis nodes, which are critical barriers for FHB spread in wheat, ROS were induced by chitin. In addition, the authors described that different defense gene expressions occurred in rachis nodes and wheat heads treated with chitin or infected with *Fusarium graminearum*, the most important causal agent of FHB. These results highlight wheat tissue-specific immune responses triggered by chitin.

An environmentally friendly approach to decrease the risk of yield losses and mycotoxin contamination is to use wheat cultivars with stable resistance to FHB. In this context, Yan et al. evaluated FHB resistance in more than 400 wheat lines with natural infection in different locations. Cultivation area and variety had an important influence on FHB and mycotoxins accumulation. Considering climatic elements, rainfall and relative humidity were key factors linked with FHB severity.

Some *Fusarium* spp. can co-occur with other fungal pathogens in host tissue. This is the case of *Fusarium pseudograminearum* and *Rhizoctonia cerealis*, causal agents of *Fusarium* crown rot (FCR) and sharp eyespot of common wheat, respectively. Up to date, there has been no information on the resistance of wheat against these two pathogens. In the contribution of Qi et al., TaWAK-6D, a wall-associated kinase (WAK) encoded by a gene located on chromosome 6D, was identified as able to confer resistance to both *F. pseudograminearum* and *R. cerealis* infection.

In conclusion, the collection of articles on this Research Topic shows the dense network of relationships that occur during *Fusarium*-plant interactions. The collected contributions have highlighted the key factors that surely will contribute to the control of this important group of plant pathogens.

## Author contributions

All authors listed have made a substantial, direct, and intellectual contribution to the work and approved it for publication.

## Acknowledgments

The editors would like to thank all reviewers who evaluated manuscripts for this Research Topic and Dr. S. Tjamos to edit an article on this Research Topic.

## Conflict of interest

The authors declare that the research was conducted in the absence of any commercial or financial relationship that could be construed as a potential conflict of interest.

## Publisher’s note

All claims expressed in this article are solely those of the authors and do not necessarily represent those of their affiliated organizations, or those of the publisher, the editors and the reviewers. Any product that may be evaluated in this article, or claim that may be made by its manufacturer, is not guaranteed or endorsed by the publisher.

## References

[B1] Escrivá L. Font G. Manyes L. (2015). *In vivo* toxicity studies of *Fusarium* mycotoxins in the last decade: A review. Food Chem. Toxicol. 78, 185–206. doi: 10.1016/j.fct.2015.02.005 25680507

[B2] Leslie J. F. Summerell B. A. (2013). “An overview of *Fusarium*”, in Fusarium: Genomics, molecular and cellular biology. Eds. Brown D. W. Proctor R. H. (Norfolk: Caister Academic Press), 1–9.

[B3] Ma L. Geiser D. M. Proctor R. H. Rooney A. P. O’Donnell K. Trail F. . (2013). *Fusarium* pathogenomics. Annu. Rev. Microbiol. 67, 399–416. doi: 10.1146/annurev-micro-092412-155650 24024636

[B4] Ma L. van der Does H. C. Borkovich K. A. Coleman J. J. Daboussi M. Di Pietro A. . (2010). Comparative genomics reveals mobile pathogenicity chromosomes in *Fusarium* . Nature 464, 367–373. doi: 10.1038/nature08850 20237561 PMC3048781

[B5] Nganje W. E. Bangsund D. A. Leistritz F. L. Wilson W. W. Tiapo N. M. (2004). Regional economic impacts of *Fusarium* head blight in wheat and barley. Rev. Agric. Econ. 26, 332–347. doi: 10.1111/j.1467-9353.2004.00183.x

[B6] Niu X. Yang G. Lin H. Liu Y. Li P. Zheng A. (2021). A novel small cysteine-rich effector, RsSCR10 in *Rhizoctonia solani* is sufficient to trigger plant cell death. Front. Microbiol. 12. doi: 10.3389/fmicb.2021.684923 PMC8421026 34497591

[B7] Pereira E. Vázquez de Aldana B. R. San Emeterio L. Zabalgogeazcoa I. (2019). A survey of culturable fungal endophytes from *Festuca rubra* susp. *pruinosa*, a grass from marine cliffs, reveals a core microbiome. Front. Microbiol. 9. doi: 10.3389/fmicb.2018.03321 PMC6343541 30700985

[B8] Segura-Mena R. A. Stoorvogel J. J. García-Bastidas F. Salacinas-Niez M. Kema G. H. Sandoval J. A. (2021). Evaluating the potential of soil management to reduce the effect of *Fusarium oxysporum* f. sp. *cubense* in banana (Musa AAA). Eur. J. Plant Pathol. 160, 441–455. doi: 10.1007/s10658-021-02255-2

[B9] Tariqjaveed M. Mateen A. Wang S. Qiu S. Zheng X. Zhang J. . (2021). Versatile effectors of phytopathogenic fungi target host immunity. J. Integr. Plant Biol. 63, 1856–1873. doi: 10.1111/jipb.13162 34383388

[B10] Viljoen A. Ma L. Molina A. B. (2020). “*Fusarium* wilt (Panama disease) and monoculture in banana production: Resurgence of a century-old disease”, in Emerging plant diseases and global food security. Eds. Ristaino J. B. Records A. (St. Paul, Minnesota, USA: APS Publications), 159–184. doi: 10.1094/9780890546383.008

